# Framing Metrics, Outcomes, and Assessment Overview in Leadership Programs

**DOI:** 10.1002/yd.70007

**Published:** 2025-08-20

**Authors:** Lindsay J. Hastings, David Rosch

**Affiliations:** ^1^ Department of Agricultural Leadership Education, and Communications University of Nebraska‐Lincoln Lincoln Nebraska USA; ^2^ Agricultural Leadership, Education, and Communications Program University of Illinois at Urbana‐Champaign Champaign Illinois USA

## Abstract

In this article, we discuss the need for the field of leadership education (LE) to employ detailed outcomes for assessing its learning interventions, along with rigorous metrics in which to measure such outcomes. We define assessment, outcomes, and metrics, and provide numerous examples of how each can be built and improved upon across a variety of leadership programs and courses. Lastly, we provide a number of specific methodological suggestions for leadership educators to consider implementing in their programs that represent quality practices in assessing leadership learning and leader development.

## 1 Introduction

Leadership education (LE) is a big business. Although $50 billion is spent annually on leader development programs within professional companies (Feser et al. 2017), most colleges and universities list the development of their students as emerging leaders as one of their primary educational goals (Kniffin et al. 2020; Rosch and Allen 2024). Yet, a strain of scholarship has existed for over a decade editorializing the sorry state of evidence that such effort, resources, and attention on LE are making a difference for the students who participate in LE programs and services. Owen's (2012, 20), writing to introduce a new national‐scale institutional survey of leadership growth in university students, stated, as a field, LE, “…faces the awkward adolescent phase where there is incongruity between what is known about effective leadership education and what is enacted in programs.” 10 years later, little progress has been made (Leroy et al. 2022).

This journal issue is designed, in part, to help address such a lack of progress. Previous articles in this issue have focused on the theory and potential examples for developing and applying a conceptual framework to LE programs (Benavides 2025). Other articles focus on the content of LE programs and ensuring alignment with chosen frameworks. Further articles focus on the learners within LE programs and how such learners are central to ensuring alignment across the overall framework, program content, and learning process (Jimenez Luque 2025). This article will focus on the part of ILA's *General Principles for Leadership Programs* (2021) that essentially asks, “Is any of this work actually making a difference?”

Before we describe theories and best practices for building strong metrics and outcomes and how to assess them, however, we first feel we need to focus on two often‐unspoken attributes of many LE programs in the education sector, and how these attributes are directly related to the laments described above. Critically analyzing these attributes helps describe *why* such a lack of progress has been made to comprehensively describe their intended outcomes, define metrics for measuring their outcomes, and build a rigorous program assessment to ultimately evaluate them.

The first attribute is the embedded nature of how students participate in LE programs while in school. Educational institutions often create and offer LE initiatives that are—admirably—open to students from diverse backgrounds, with diverse prior LE experiences, and with diverse personal and career goals (Reyes et al. 2019). LE programs in schools and universities, essentially, attempt to help students on their personal and professional trajectories without being able to control—or often even know—what those trajectories have been or what they hope to be in the future. Although such openness and inclusivity are certainly strengths, they become a weakness when attempting to define realistic concrete outcomes for LE programs that are more than overly broad and undefined statements like, “Students will grow in their self‐awareness.” Amidst such diversity, the most efficient way to create a structure of assessment is to leave the defining of outcomes and metrics up to the individual student (e.g., “How significant was your learning as a result of participating in this LE program?”). In such an assessment environment, the definition of “leadership development” becomes something that rests in the eye of the beholder.

The second, often invisible, attribute that damages the ability to rigorously assess LE initiatives in educational contexts is that such initiatives are situated both as spaces for growth AND spaces to spotlight (e.g., for prospective student recruitment and funding). If a university's “President's Leadership Program” produces assessment data that suggests that the initiative may not be as impactful as hoped for, what does that imply about the President? Or the staff who are responsible for building and delivering it to students? Amidst these potentially conflicting priorities, LE assessment efforts are, perhaps subconsciously, specifically designed to report unsurprising growth and learning in students.

Taken together, both attributes reinforce the other. The result is that most LE programs possess weakly defined outcomes, self‐reported and ambiguous metrics, and a structure of assessment designed to almost undoubtedly report positive information about their impact. Seen from this perspective, the complaints described above seem to make more sense. With these challenges now made explicit, the remainder of the chapter will describe the current state of research focused on evaluating LE efforts and suggestions for applying evidence‐based practices in building LE outcomes assessment structures.

Although our understanding of impact continues to lag despite the growing footprint of LE programs and participants (Rosch and Hastings 2022), advancements in the field of leadership have taught us several things, namely:

- The importance of undergirding leader and leadership development (LD) in a conceptual framework that matches the complexity and needs of the leadership learner (Avolio et al. 2009; Beatty et al. 2022; Day and Liu 2019; Hastings et al. 2023; McKee 2022)
- Leadership is not just about the leader at a static moment in time, but rather, is dynamic and involves all voices in its dynamic process (Day et al. 2014; Komives et al. 2013; Komives and Owen 2023; McCusker et al. 2019)
- LD is longitudinal, non‐linear, multi‐level (both individual and team levels), happening earlier than we think, and largely influenced by context; thus, LE does not serve its intended purpose well via disconnected short‐term programs devoid of learner context and focused solely on leader development (Diaz et al. 2022; Hastings et al. 2023; Martin et al. 2022; Liu et al. 2021; Lord 2019; Murphy 2019; Murphy and Johnson 2011; Sessa et al. 2023; Yammarino and Dionne 2019).

Despite these advancements, we still do not know much about the trajectories of student LD, factors external to training that shape development, *how* LD occurs, and the effects of leadership training on team dynamics (Rosch and Wilson 2022). These advancements not only challenge the design of leadership programs but also their metrics, outcomes, and assessment. Through this article, we address ILA's *General Principles* (2021) related to metrics, outcomes, and assessment, shaped by two guiding questions:

1. How can we craft program outcomes that reflect intended changes in dynamic leadership processes, long‐term manifested behavioral change, and individual and collective levels?
2. How can we advance our metrics and methods to assess these intended changes to reflect their long‐term, non‐linear, multi‐level, dynamic, interdependent, complex, and contextually laden nature?

## 2 Explanation of Metrics, Outcomes, and Assessments

Metrics, outcomes, and assessment are all interrelated. Stated program outcomes ultimately drive the plan for assessment, and its metrics are the mechanism through which the assessment plan is successfully executed (McElravy 2022). Below, we will offer broad definitions of metrics, outcomes, and assessment and emphasize their relation to leadership learning. We begin our explanation with a program's “outcomes,” which are the ultimate determinant in any efforts to determine the effectiveness and overall success of LE efforts, following with explanations for “assessment” and then “metrics” that can be applied to evaluate outcomes. We recognize our ordering of concepts differs from how they are listed in ILA's Guiding Principles document but believe that our ordering provides for a more coherent way to explain how each are related. We will return to this order later in the article when we describe how each might be applied.

### 2.1 Outcomes

Outcomes illustrate the measurable impact of our leadership programs and include both the level of the leadership learner as well as the program itself (Patterson et al. 2017). The ILA *General Principles* document highlights that program outcomes need to be explicit, transparent, and concrete and need to be regularly assessed for program improvement as well as for accountability to stakeholders. The *ILA General Principles* document further highlights that program outcomes can be cognitive, affective, and/or behavioral and should explicate the program's definition of leadership, the contexts for which program participants are being prepared to impact, and the specific goals of the program itself. *Everything* should be driven by program outcomes, including program design, learning objectives, pedagogical methods, recruitment strategies, and an assessment plan. We cannot overstate that the more detailed and comprehensive these outcomes are, the easier and more straightforward it will be to investigate the impact of the initiatives (Rosch and Wilson 2022).

### 2.2 Assessment

Assessment embodies the plan through which we determine whether or not the program has met its intended outcomes. Assessment, evaluation, and research are not the same thing (DeSawal and Peck 2022), so be careful about conflating them. *Assessment* is the data we collect and use to indicate the accomplishment (or lack thereof) of program outcomes. *Evaluation* places value on the assessment data to determine the effectiveness of the program in meeting its intended outcomes and to drive program improvement efforts. *Research* is when we utilize leadership programs and/or program participants to investigate broader concepts in the leadership field. Ideally, assessment and evaluation cycles should be consistent with program delivery cycles and should inform program improvement, both in terms of stated outcomes as well as program design (Patterson et al. 2017). No single right method exists for assessing outcomes. The assessment plan should be explicitly matched to the program outcomes so that the assessment data provide meaningful information (McElravy 2022).

### 2.3 Metrics

Metrics are enfolded within an assessment plan and are the mechanisms through which we determine whether or not a program has met its intended outcomes. Ultimately, we want the metrics, outcomes, and assessments to be interrelated. So, ideally, program outcomes are articulated in a way that foreshadows the metric through which it will be assessed, and metrics are mapped to the assessment plan, which is mapped to the program outcomes (Patterson et al. 2017). Metrics help to answer the question, “How will we know if the outcome has been met? At what threshold will we say, ‘We've done it!!’?”

Numerous potential pathways exist for putting these concepts into action in one's program evaluation plan (Hoole and Martineau 2014). Later on, we describe these concepts using practical guidelines and examples. A subsequent chapter in this Issue is also dedicated to describing concrete examples for integrating these ideas into an effective evaluation plan. Before we dive deeper, however, we want to focus on how the LE field has historically applied outcomes, assessment, and metrics, and what we can learn from such history.

### 2.4 Making Assessment More Than an Afterthought

Largely, the reason why our understanding of the LE footprint has lagged is that assessment and evaluation have often been an afterthought, if a thought at all, for program architects (Leroy et al. 2022). Even if we (and we, the authors, include ourselves in this “we”!) did think about assessment, we, like electrical currents, often followed the path of least resistance. We surveyed participants on the last day (or hour) of the initiative—rather than following up later after they had a chance to apply their learning. Collecting such data was quick and easy. After all, we had a captive audience and knew we could get a good response rate, and we knew, on trend, that participants would feel the most energized by the program at the very end when they have an intrinsic motivation to report that their time was not wasted (e.g., *the honeymoon effect*, Rosch and Schwartz 2009).

We likely asked about things that were easy to measure, could be measured via self‐report, and would likely be impacted by short‐term interventions (e.g., self‐confidence, motivation to lead, comfort in engaging in leader behaviors, growth in self‐awareness). As a field, we have been largely afraid to assess developed leadership capacity via manifested behavior and have relied on proxies instead (Fischer et al. 2023). In so doing, the pre/post surveys became our “Linus blanket” because, other than some aberrations with self‐efficacy, people most often say they are “better than they were” immediately following their participation in a formal program (e.g., *the horizon effect*, Rosch and Schwartz 2009).

But we can evolve! As previously discussed, advancements in LE can and should advance our leadership program outcomes, which should then advance our assessment plans and associated metrics. Table 1 below highlights several significant advancements in the leadership field that we described earlier, and how each might support evolution in our outcomes, assessment, and metrics.

**TABLE 1:** Advancements in leadership and considerations for metrics, outcomes, and assessment.

Advancements in leadership field	Outcome consideration	Assessment consideration	Metric consideration
Each leadership learner is complex, and leadership learning is influenced by context (Avolio et al. 2009; Beatty et al. 2022; Day and Liu 2019; Hastings et al. 2023)	What parts of our learners’ context inform their implicit assumptions about leadership coming into a program? How will their context influence how leadership learning is applied? How can our program outcomes reflect this acknowledgment of context? How can the leadership theories undergirding our programs explicitly match complex leadership learner needs?	Assess learner needs before articulating program outcomes (Lacerenza et al. 2017) Articulate program outcomes that acknowledge a desired behavior manifested in different ways, depending on context, to allow for more flexible assessment pathways Assessment plans might need to be flexible and co‐constructed with participants	Flexible metrics (we do not HAVE to have the same metric for every participant!!) Metrics co‐determined with participants Account for the influence of “nests” (that one individual's leadership learning pathway might be influenced by their group membership and institutional affiliation)
Leadership is a dynamic process (Day et al. 2014; Komives et al. 2013; McCusker et al. 2019)	How can our program outcomes reflect a focus on process and interdependency? Articulate program outcomes that address knowledge of process (cognitive), confidence in navigating process (affective), and behaviors that positively contribute to process (behavior)	Assessment plans might need to address the multi‐faceted (e.g., cognitive, affective, and behavioral) components of process Assessment methods will likely need to include multiple sources and multiple methods (Hoole and Martinau 2014).	Identify metrics that indicate *process* improvements (e.g., inclusion of multiple perspectives, greater number of solutions generated to any one given problem than before, satisfaction with process)
LD is longitudinal and non‐linear (Day and Liu 2019; Diaz et al. 2022; Lord 2019)	How might we articulate outcomes to indicate that the accomplishment of the outcome will not happen at the end of a program? How might we articulate outcomes that do not automatically require trajectories that are “up and to the right”?	Assessment methods will need to involve tracking an outcome over time with (ideally) three or more waves of data and a sensible time metric (the program outcome should perhaps foreshadow when we would likely see perceptible changes).	Metric considerations might involve finding an equivalent comparison group, finding valid and reliable measures, and specifying direct and indirect mechanisms. Although there are multiple statistical pathways to analyze longitudinal data, especially anticipated non‐linear trajectories (e.g., latent growth curve modeling), there can be other ways to collect and analyze longitudinal data (e.g., degree‐of‐change graphing with an analysis of trends and inflection points)
LD is multi‐level (both individual and collective levels; Martin et al. 2022; Sessa et al. 2023; Yammarino and Dionne 2019)	Outcomes should explicitly indicate if they are focused on individual leader development, interpersonal relationships, team processes and dynamics, and/or collective functioning (see Wallace et al. 2021).	Assessment methods will need to be intentionally matched to the explicitly stated level of the program outcome. Assessment methods will likely need to include multiple sources and multiple methods (Hoole and Martinau 2014).	Associated metrics will also need to be intentionally matched to the explicitly stated level of the program outcome.
LD is happening earlier than we think and is happening across the lifespan (Hastings et al. 2023; Liu et al. 2021; Murphy 2019; Murphy and Johnson 2011)	How might our leadership programs connect to earlier LD experiences for leadership learners? How might we think beyond singular, event‐based leadership programs to consider sequencing LD efforts to develop leadership learners over time alongside regular maturation processes?	Assessment methods may need to be coordinated across programs. Assessment methods will need to correspond with the spacing of the LD elements	Identify metrics that demonstrate a developmental pathway across programs Metrics will need to indicate desired changes that would be most appropriately impacted by particular LD elements.

## 3 How to Use General Principles for Metrics, Outcomes, and Assessment

ILA's *General Principles for Leadership Programs* offers the following questions related to metrics, outcomes, and assessment:

- *Does the program utilize a cycle of assessment, evaluation, and decision‐making for a continual cycle of improvement based on the program's stated outcomes?*
- *Is the assessment process for data collection clear?*
- *Do the processes for evaluation and decision‐making involve key constituencies?*
- *Does decision‐making inform continued improvement in developing curriculum and outcomes?*
- *Does the program utilize assessment and evaluation cycles for individual learning outcomes, initiatives, as well as the overall program outcomes?*
- *Does the program also take into consideration campus‐level goals and outcomes (e.g., mission, values, and vision)?*
- *Does the program consider its context within the broader society in its assessment and evaluation efforts?*
- *How does the program communicate the results of its assessment and evaluation cycles to relevant stakeholders, including participants and the general public?*

The questions posed in ILA's *General Principles* document largely follow the assessment cycle process of (a) gathering information, planning, and conceptualizing, (b) collecting and analyzing data, (c) interpreting and communicating findings, and (d) implementing changes and sharing lessons learned (Patterson et al. 2017) offer a step‐by‐step guide for how to plan and execute an LD program evaluation effort, shaped by the assessment cycle). The questions in the *General Principles* not only follow the assessment cycle process for continuous improvement but also highlight considerations of mapping program outcomes to campus‐level goals and outcomes, as well as to broader societal implications. These considerations become important tools for indicating value beyond just meeting the program outcomes themselves.

Although the *General Principles* questions offer important procedural questions for addressing the assessment cycle, evolving leadership program outcomes, assessment methods, and metrics to address advancements in our understanding of leadership might serve to produce more productive outputs from the assessment cycle process.

### 3.1 Program Outcomes: “All Roads Lead to Rome”

As discussed earlier, *everything*, and especially one's assessment plan and associated metrics, should be led by one's program outcomes. In this way, everything one measures with the mind to evaluate the success of the program should ultimately lead to its stated desired outcomes. When developing program outcomes, consider the following questions from Day et al.'s (2021) introductory article for a special issue in *Leadership Quarterly* on 21^st^‐century LD: (a) What are we developing?; (b) Who are we developing?; and (c) How does leadership develop? As highlighted in Table 1, advancements in leadership should be reflected in our evolved leadership program outcomes (i.e., acknowledging leadership learners as complex, acknowledging leadership as a process, and not solely focused on the leader). Additionally, outcomes should foreshadow assessment methods and associated metrics that acknowledge LD as longitudinal, multi‐level, non‐linear, influenced by context, and connected to other LD experiences (Hastings 2023).

### 3.2 Assessment Methods and Associated Metrics

Evolved program outcomes that reflect advancements in leadership should be explicitly matched with assessment methods and associated metrics that allow for more productive assessment cycle outputs. Below are some suggestions for how assessment methods and associated metrics might evolve, following Hoole and Martinau's (2014) summary of best practices.

#### 3.2.1 Multi‐Source

Kittens often like to see themselves as tigers in the mirror! Be careful about relying exclusively on self‐assessments. The *Hollywood Effect* in outcomes assessment is real (Rosch and Schwartz 2009)—participants will often face difficulty in objectively measuring themselves. We need to refrain from relying purely on self‐report data.

#### 3.2.2 Multimethod

Triangles are important to more than just pyramids! Validity within outcomes assessment can be enhanced via triangulation, which can also provide a fuller picture for stakeholders. Thus, consider which forms of data best serve the identified metrics. For example, how might qualitative methods enhance our ability to measure change within complex leadership processes (see Kniffin and Priest 2022)? If multiple forms of data will be required to assess a particular outcome, be intentional about a mixed methods approach to data collection and analysis (see: Hastings 2022).

#### 3.2.3 Longitudinal

Sustained LD is not a short‐term endeavor! LD is rarely accomplished by short‐term initiatives focused on attitudinal or awareness changes in participants (even as these short‐term changes might be necessary for more long‐term development). Thus, consider how a longitudinal approach might be threaded through program outcomes, assessment plans, and associated metrics. For example, it might be important to ask questions in the program design phase, such as: (a) What long‐term changes do we hope for? (b) How might we reasonably assess change over time?; and (c) What type of change over time would we determine as “successful” given the program outcome?

#### 3.2.4 Psychometric Measurement

Psychometrics—the quantitative field of psychology dedicated to outcomes, metrics, and assessment—can be helpful here! Validity and reliability of a quantitative outcomes assessment can be enhanced by utilizing scales that have demonstrated psychometric properties. Try to find measures of program outcomes that have demonstrated validity and reliability, and therefore have broad value, in addition to self‐identified indicators, which may be more appropriate for understanding unique contexts. Undergirding leadership programs with a conceptual framework that best matches leadership learner needs can point to appropriate psychometric assessments. We urge the use of creative thinking here.

Consider imaginative ways to measure changes in manifested behavior after participants have had time in their “natural habitat.” For example, a few weeks after an initiative has concluded, consider asking its participants to describe the frequency of desired leader behaviors within a concrete (and short) timeline. This can be accomplished by inviting responses to items such as, “How many times within the past week have you… (desired behavior): never, once, a few times…”

#### 3.2.5 Isolating Effects

Specificity is key! The more specific our program goals and learning objectives, the more reliably we can evaluate changes in participants over time as related to the desired impact of the leadership program. Although acknowledging the influence of context, consider what we might reasonably expect to be changed uniquely by participating in one's leadership program. This exercise may help us to refrain from articulating program outcomes that indicate broad changes such as ‘enhanced leadership capacity’ (e.g., *Hallmark effect*, Rosch and Schwartz 2009) or overstating the effects of spending an afternoon in a leader development workshop.

## 4 Conclusion

We began by highlighting the somewhat disappointing state of program evaluation related to leader and LD initiatives across educational contexts and explained the two factors that fundamentally contribute to this state—our desire to serve a diverse set of students with a diverse set of needs, and the press to show the success of the LD initiatives for which we are responsible. To help address these factors, it is imperative for program architects to connect their assessment efforts with the prior Guidelines.

Because “all roads lead to Rome” with outcomes, program outcomes are shaped by and indicate desideratum for their *Context* (see: Guthrie and Devies 2025), are ideally undergirded by a *Conceptual Framework* (see: Roberts 2025) that matches leadership learner needs, inform the design of *Content* and *Learning* (see Jimenez‐Luque 2005; Jenkins and Rocco 2005), and stated program outcomes ultimately drive the plan for assessment and indicate the mechanism (i.e., the *Metric*) through which the program success is achieved.

Evolving our leadership program outcomes to articulate intended changes in dynamic leadership processes should include the factors we describe above: a focus on long‐term manifested behavioral change, including individual and collective levels of analysis, and evolved metrics and methods to assess these intended changes. Further, they should incorporate an understanding that the leader and LD is non‐linear, multi‐level, dynamic, interdependent, complex, and contextually laden. Doing so will allow us to have more productive outputs from assessment cycle processes that help us to understand the impact of our growing LE footprint.
